# mRNA Targets of RNA-Binding Proteins Suggest an Extensive System for Post-Transcriptional Regulation

**DOI:** 10.1371/journal.pbio.0020082

**Published:** 2004-03-16

The single-celled Saccharomyces cerevisiae, commonly known as baker's yeast, measures just 2 microns—it takes about 4 billion to fill a teaspoon. But as a eukaryote (its cells have nuclei), its genes function in much the same way a human's do. For a gene to function, its DNA sequence must first be transcribed into RNA (called messenger RNA, or mRNA), whose sequence can then be translated into a specific string of amino acids to form the unique protein that the gene encodes.

The population of mRNA transcripts in each cell (its “transcriptome”) is dynamic—the genome uses its vocabulary of genes to write an ever-evolving script for the cell as its life unfolds and its environment changes. By binding to specific sequences of DNA, proteins called transcription factors process signals from the cell's sensory and information-processing systems to control which genes are transcribed in each cell, under what conditions, and at what rate. While the steps and regulatory programs that govern gene expression at this level are reasonably well known, much less is known about the orchestration of the later steps in the gene expression program—where in the cell each mRNA molecule goes when it leaves the nucleus, at what rate and under what conditions it is translated into protein, and how long it survives.[Fig pbio-0020082-g001]

**Figure pbio-0020082-g001:**
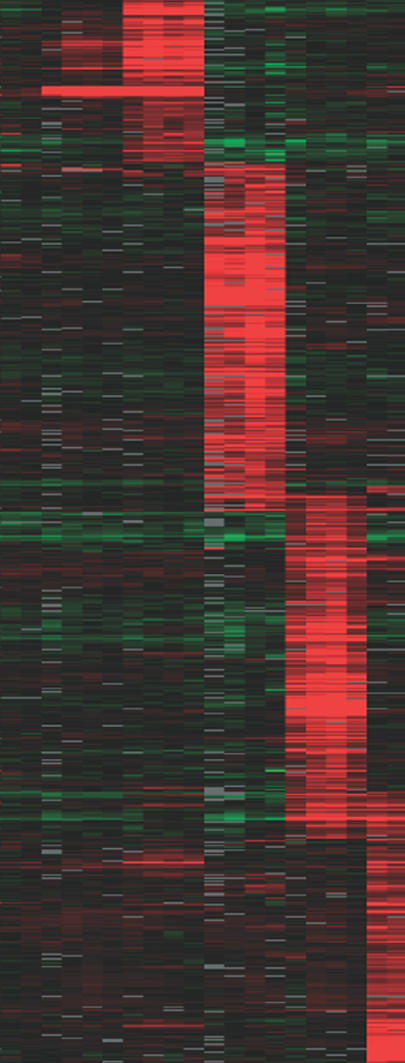
Cluster of RNA targets for Puf proteins

RNA-binding proteins (RBPs) have been implicated in diverse aspects of post-transcriptional gene regulation. Hundreds of RBPs are encoded in the eukaryotic genome, but because few have been studied in detail and few of their mRNA targets are known, the nature and extent of an RBP-mediated post-transcriptional program has been obscure. Now a systemic analysis of a specific family of RBPs and their mRNA targets in yeast by André Gerber, Daniel Herschlag, and Patrick Brown, of Stanford University, suggests that such a program may exert detailed control over the life history of every mRNA. By selectively binding and regulating specific classes of mRNAs, RBPs may provide a mechanism to coordinate the collective fate of these transcripts and serve as an integral part of the global transcriptome.

Gerber, Herschlag, and Brown focused on the binding targets of a family of RBPs called Pumilio-Fbf (Puf) proteins, which are defined by the presence and configuration of an amino acid domain that mediates RNA-binding. Little is known about the physiological function of the five yeast Puf proteins the researchers studied here (called Puf1p-Puf5p). After using “affinity tags” to snag each of the five Puf proteins from yeast cells, together with their bound mRNA targets, the researchers identified the associated mRNAs with microarray analysis. They found more than 700 mRNAs bound by at least one Puf protein, with each Puf RBP targeting a distinct group of mRNAs. The group of mRNAs associated with each Puf protein turned out to encode proteins with strikingly similar functions and locations in the cell. Many of the mRNA sets encode proteins that reside in the same cellular location, are part of the same protein complexes, or act in the same signaling pathway. Some Puf proteins target mRNAs that encode membrane proteins while others preferentially bind to mRNAs that encode proteins involved in cell division. The most pronounced bias occurs with Puf3p, which overwhelmingly binds mRNAs that encode proteins destined for the mitochondria, the cell's power generators.

This selective tagging of functionally related mRNAs by specific RBPs suggests a mechanism for coordinated global control of gene expression at the post-transcriptional level. Just as transcription factors regulate transcription by binding to specific DNA sequences, RBPs may mediate regulation of the subcellular localization, translation, and degradation of the set of specific mRNAs they target. Noting the striking themes in the subcellular localization of the proteins encoded by the mRNAs bound by each Puf protein, Gerber, Herschlag, and Brown propose that RBPs may play important roles in the subcellular localization and efficient assembly of protein complexes and functional systems by ensuring that the location in the cell at which mRNAs are translated “is not left to chance.” Since the number of RBPs encoded in eukaryotic genomes approaches that of transcription factors, the regulatory program that controls the post-transcriptional fate of mRNAs—their localization, translation, and survival—may prove to be nearly as diverse and complex as the regulation of transcription itself.

